# Occlusal force analysis with T-Scan and Innobyte in adult patients: a prospective study

**DOI:** 10.1038/s41598-026-51891-3

**Published:** 2026-05-07

**Authors:** Mauro Lorusso, Fariba Esperouz, Elena D’Angelo, Michele Tepedino, Angela Pia Cazzolla, Lorenzo Lo Muzio, Lucio Lo Russo, Domenico Ciavarella

**Affiliations:** 1 Department of Clinical and Experimental Medicine, Dental School of Foggia, University of Foggia, Foggia, Italy; 2 Department of Biotechnological and Applied Clinical Sciences, Dental School of L’Aquila, University of L’Aquila, L’Aquila, Italy

**Keywords:** T-Scan, Innobyte, Digital occlusal analysis, Vertical growth, Centre of force, Occlusal force, Diseases, Health care, Medical research

## Abstract

This prospective study investigated differences in occlusal force among adult patients with various vertical skeletal patterns and evaluated the association between total occlusal force and the centre of force position. Ninety-six subjects (46 females, 50 males; mean age 26.4 years, SD 1.4) with Angle Class I malocclusion were enrolled and classified as hyperdivergent, normodivergent, and hypodivergent according to the SN-MP angle. Digital occlusal analysis was performed using the T-Scan Novus system to assess occlusal contact time and centre of force position at maximum intercuspation, while maximum occlusal force was recorded using the Innobyte device. The centre of force was classified as anterior, centred, or posterior based on its spatial location within the dental arch. Intergroup comparisons were conducted using Welch ANOVA with Games-Howell post hoc tests, and a generalized linear model evaluated the association between occlusal force and centre of force position (α = 0.05). Hypodivergent subjects showed the highest total occlusal force and occlusion time (513.3 N, 7.9 s), while hyperdivergent individuals exhibited the lowest (399.54 N, 6.93 s). In hypodivergent subjects, the centre of force was mainly anterior (70.7%), whereas in hyperdivergent individuals it was predominantly posterior (76.41%). A significant association was observed between total occlusal force and centre of force position (*p* < 0.001). Pairwise comparisons showed higher occlusal force (+ 54.61 N, *p* < 0.05) associated with an anterior centre of force (+ 116.78 N; *p* < 0.001) compared with the posterior group. Overall, increased occlusal force correlated with an extended time required to reach maximum intercuspation.

## Introduction

 Occlusal force analysis is a fundamental aspect of orthodontic and prosthetic treatment planning. Previous studies have demonstrated that occlusal function cannot be fully understood by static evaluation of dental arches and supporting skeletal structures alone, as mastication depends on the dynamic interaction among teeth, jaws, masticatory muscles, and surrounding soft tissues, including lips and cheeks^1^. For this reason, assessment of occlusal forces has been widely recognised as an important indicator of functional balance within the stomatognathic system.

Optimization of masticatory function is a fundamental objective of orthodontic treatment, particularly in patients with complex malocclusions. Modifications of occlusal contacts can significantly influence masticatory dynamics and neuromuscular activity, requiring careful planning to achieve a new functional equilibrium^2,3^. This consideration is especially relevant in cases involving dental impactions, where alterations in occlusal relationships may affect both force distribution and functional stability^4,5^. Previous investigations have also highlighted the importance of evaluating changes in the occlusal plane with respect to facial growth pattern and dental arch morphology^6^. Furthermore, gradual introduction of occlusal changes has been recommended to allow physiological adaptation of the stomatognathic system, thereby minimising the risk of periodontal overload and temporomandibular joint dysfunction^7,8^.

Several diagnostic tools have been introduced to assess occlusal force and occlusal dynamics. Among them, the T-Scan system is widely used to obtain quantitative information on occlusal contact distribution, centre of force (COF), intercuspation timing, and the relative distribution of forces between hemiarches^9^. The system also permits evaluation of the occlusal centre of gravity, defined as the point at which the resultant of all occlusal forces is applied. From a functional standpoint, the localization of this point is relevant to the interpretation of neuromuscular coordination and the maintenance of occlusal balance and functional stability^10^. Nevertheless, despite its ability to characterize occlusal dynamics in detail, the T-Scan system does not provide absolute measurements of occlusal force magnitude^10^. In contrast, the Innobyte system is a medical device developed for the measurement of maximum occlusal force, with output expressed in Newtons, both unilaterally and across the entire dental arch^11^. The system consists of a handheld measuring unit connected to a fluid-filled intraoral mouthpiece, which allows force transmission to internal sensors for real-time recording. Therefore, the combined use of T-Scan and Innobyte may provide complementary information, integrating the assessment of occlusal dynamics and force distribution with absolute force measurement.

Accurate measurement of occlusal force is complex, because it is modulated by several factors, including: craniofacial morphology, dental contact patterns, occlusal scheme, and the presence of malocclusions or parafunctional habits. These variables must be carefully considered in order to accurately interpret occlusal data in both clinical and research settings^11^.

Occlusal forces are not uniformly distributed along the dental arch, but instead follow a biomechanical gradient shaped by occlusal anatomy, which is the pattern of tooth contacts, and the functional dynamics of the masticatory system. Evidence from the literature indicates that the region of Maximum Occlusal Load (MOL) is typically localised at the mesiopalatal cusp of the maxillary first molars^12^. The magnitude and distribution of occlusal forces are influenced by specific occlusal characteristics, which determine the number, location, and quality of tooth contacts. A physiologically balanced occlusion promotes biomechanically efficient force distribution, whereas, malocclusions can compromise these dynamics by reducing occlusal forces during mandibular elevation, potentially compromising masticatory efficiency^13,14^.

Recent evidence supports an association between craniofacial morphology, particularly vertical divergence and masticatory muscle characteristics that may influence occlusal force generation. A systematic review and meta-analysis^15^ reported that hyperdivergent individuals present significantly reduced masseter muscle dimensions compared with normodivergent and hypodivergent subjects, suggesting a morphological substrate for lower force capacity across vertical skeletal patterns. Consistently, clinical studies evaluating maximum bite force across facial morphologies have reported differences attributable to vertical pattern classifications and related cephalometric features^16^. In addition, skeletal indicators linked to vertical morphology, such as the gonial angle, have been shown to correlate inversely with maximum occlusal force in posterior regions, reinforcing the biomechanical relationship between craniofacial form and occlusal loading^17^. Beyond force magnitude, computerized occlusal analysis has enabled objective assessment of occlusal force distribution and centre of force parameters under different functional and occlusal conditions, providing a reliable tool for quantitative analysis^18^. However, evidence linking these parameters to craniofacial growth patterns remains limited.

Despite the recognised importance of occlusal loading in clinical diagnostics and treatment planning, the quantitative relationship between occlusal force magnitude and skeletal divergence remains insufficiently investigated. Furthermore, the association between occlusal force magnitude and the spatial position of the centre of force remains unclear. A clearer understanding of these relationships is essential for comprehensive functional evaluation of the stomatognathic system and for improving orthodontic treatment planning. While various factors influence occlusal loading, including skeletal and arch morphology^6^, muscle function, and dental occlusion, the relationship between craniofacial growth patterns and occlusal force remains insufficiently explored. Therefore, the aim of the present prospective study was to analyze occlusal force in adult patients using the T-Scan and Innobyte systems, in order to investigate whether different craniofacial growth patterns are associated with variations in occlusal force magnitude and to evaluate the correlation between occlusal force and centre of force position.

## Materials and methods

All procedures described in this research protocol were conducted in accordance with the Declaration of Helsinki and received approval from the Ethics Committee of the University of Foggia. (approval number 46/CE/2025). This study complies with the Strengthening the Reporting of Observational Studies in Epidemiology (STROBE) guidelines for observational research^19^.

Ninety-six participants (46 females and 50 males), with a mean age of 26.4 years (SD 1.4) were enrolled based on the following inclusion criteria: Angle Class I malocclusion and a symmetrical dental arch. Exclusion criteria were: the presence of dental agenesis; periodontal disease; loss of one or more teeth; extensive dental restorations; fixed or removable prostheses; temporomandibular joint (TMJ) disorders; and current or previous orthodontic treatment. All study participants were healthy adults, and all were undergraduate students at the University of Foggia, who were prospectively enrolled after signing an informed consent form to participate. Participants were prospectively enrolled from 01/2025 to 09/2025.

Study participants were divided into three groups according to their facial divergence. To classify subjects according to vertical divergence, the SN-MP angle cut-off values reported by the Italian Board of Orthodontics (IBO) and the European Board of Orthodontics (EBO)^20^ were used:

- Hyperdivergent subjects: SN-MP > 35.5°.
- Normodivergent subjects: 30.5 ≤ SN-MP ≤ 35.5°.
- Hypodivergent subjects: SN-MP < 30.5°.

The cephalometric characteristics of the study sample are listed in Table 1.

**Table 1:** Cephalometric data of the sample.

Sample	Divergence angle(SN-MP)	Standard deviation	Minimum	Maximum
Hyperdivergent (15 F, 17 M *n* = 32)	36.1	0.4	34.4	37.6
Normodivergent (16 F,16 M *n* = 32)	32	0.5	30.6	33.1
Hypodivergent (15 F,17 M *n* = 32)	29.4	0.6	26.9	30.2

A priori power analysis was performed using G*Power (version 3.1.9.2; Franz Faul, Universität Kiel, Germany). To detect an effect size of 0.4 using a one-way Anova test with an alpha level of 0.05 and a statistical power of 0.90 (1 − β), a minimum sample size of 28 participants per group was required^21^. The measurements were carried out at the university dental clinic by two of the authors, both experienced orthodontists. To minimize random errors, cephalometric measurements were repeated after a 15-day interval by a single calibrated examiner. The random error was calculated using Dahlberg’s formula (*S* =√∑ *d* ^2^/2*N*), where *d* is the difference between the first and second measurements and *N* the number of radiographs evaluated^22,23^. The random error ranged between 0.2 and 0.3 degrees.

### Measurement protocol

The T-Scan Novus system (Tekscan Inc., South Boston, MA, USA) was used for digital occlusal analysis^24^. The system includes a handpiece connected to a computer via USB and a support base for mounting interchangeable piezoelectric sensor foils, 100 μm in thickness and available in various sizes. The sensor is pressure-sensitive and records occlusal forces by detecting changes in electrical resistance during tooth contact. Data acquisition occurs in real time, allowing the system to record key parameters, including the timing, location, contact distribution, and relative force percentages of occlusal contacts. This enabled a detailed, dynamic evaluation of occlusal function during mandibular closure into maximum intercuspation.

Occlusal analysis was first performed using the T-Scan Novus system (Tekscan Inc., Boston, MA, USA), a computerized device that enables dynamic recording of occlusal contacts. entence for the paper:

All recordings were obtained with participants seated upright in the dental chair, with the head maintained in a relaxed and supported position. This standardized upright posture was adopted to approximate a physiological functional condition, clinically comparable to the alert feeding position, thereby better reflecting real-life masticatory function while reducing postural variability and enhancing measurement reproducibility.

For each subject, a sensor of appropriate size was selected based on the individual’s specific dental arch morphology. The sensor was accurately positioned between the upper and lower central incisors, ensuring that the handle remained parallel to the occlusal plane throughout the recording process. The sensor was placed intraorally, and the patient was instructed to occlude in maximum intercuspation (MI). An initial single recording of approximately 5–7 s was captured, followed by three consecutive recordings to ensure reproducibility. The T-Scan software enabled dynamic analysis of occlusal function, allowing for the evaluation of the following parameters:

1. Centre of force: defined as the spatial position of the occlusal centroid during MI, and categorised as anterior, central, or posterior.
2. Occlusion time: defined as the duration (in seconds) from the initial occlusal contact to the software-identified maximum intercuspation point (MI; point B), corresponding to the moment at which maximum force distribution was reached. The software automatically calculated this parameter as the time interval between the first detected contact and that reference point.

The position of the centre of force (COF) was determined during the bite registration phase, at maximum intercuspation, ensuring full engagement of the teeth in their natural occlusal relationship. Using the T-Scan system, the COF was recorded as the point where the resultant occlusal load was concentrated. Based on its spatial location relative to the dental arch, the COF was classified into one of three categories:

- Anterior: indicating that the COF was localised toward the anterior region (incisor/canine area) of the dental arch;
- Centred: indicating that the COF was located precisely at the geometric centroid of the occlusal contact area;
- Posterior: indicating that the COF was localised toward the posterior region (premolar/molar area) of the dental arch.

**Fig. 1:**
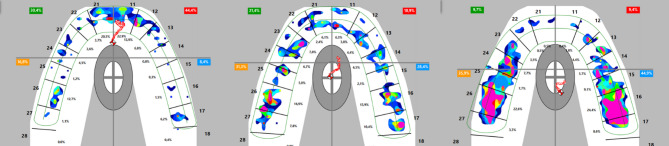
Heat map images illustrating the position of the centre of force (COF): anterior (left, hypodivergent), centred (center, normodivergent), and posterior (right, hyperdivergent).

Figure 1 illustrates the centre of force position across the three study groups. As shown in Fig. 1, the COF was categorized according to its spatial location relative to the dental arch. This classification was based on the software-generated display and not on manually predefined spatial thresholds.

Before the recordings, the sensitivity level of the T-Scan sensor was adjusted for each participant using the software’s built-in sensitivity setting procedure, in order to match the subject’s bite force range and avoid signal saturation. Recordings were accepted only after an adequate sensitivity setting had been achieved. In addition, sensor size and positioning were standardized for all participants according to the dental arch morphology and the manufacturer’s instructions.

The Innobyte [Kube Innovation Inc.] system is a medical device designed to measure maximum occlusal force, both unilaterally and across the entire dental arch^11^. It consists of two main components: a handheld measuring unit and a fluid-filled mouthpiece. The device is capable of recording occlusal forces ranging from 0 to 2000 N, with a measurement accuracy of ± 5%. When the patient bites down on the mouthpiece, the applied force is transmitted uniformly through the fluid medium, allowing for accurate detection by internal sensors in the instrument. The measured force values are displayed in real time on the LED screen of the main unit, with output provided in Newtons. Each reading provides the total occlusal force, as well as the occlusal force distribution between the left and right sides of the arch.

Before data acquisition, the Innobyte device was checked for proper functioning and used under standardized recording conditions according to the manufacturer’s instructions. The same sensor positioning, participant posture, and measurement sequence were applied for all subjects to improve consistency and reproducibility. For each measurement, participants were seated upright in the dental chair, with the head held upright and parallel to the floor.

**Fig. 2:**
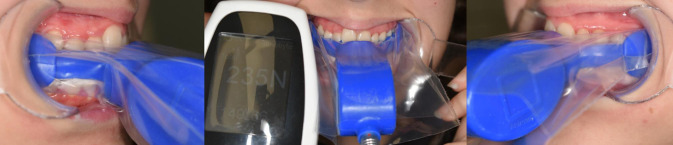
Measurement of bite force using the Innobyte device.

For each measurement, the recording sensor was positioned intraorally between the dental arches, and the central notch was aligned with the patient’s maxillary and mandibular central incisors. Data acquisition was initiated by activating the control button on the handpiece, and the patient was instructed to perform a maximal voluntary clench. Upon completion of the force recording, the mouthpiece was carefully removed, and the patient was asked to swallow. After swallowing, the patient was told to maintain a relaxed mandibular rest position for 10 s before the subsequent measurement. To ensure data consistency and minimize intra-individual variability, a multibite scan protocol was implemented, consisting of three consecutive bite recordings per study participant. For statistical analysis, the mean of the three repeated measurements was used. During measurements, the standardized thickness of the mouthpiece positioned between the dental arches prevented physiological occlusion by maintaining a controlled interocclusal separation. Figure 2 shows the Innobyte device used for the measurements.

### Statistical analysis

Data were analysed using GraphPad Prism software 6.0 (GraphPad Prism Software, San Diego, CA, USA). The primary null hypothesis stated that no significant correlation existed between facial growth pattern and occlusal force; the secondary null hypothesis stated that no significant correlation existed between occlusal force and the position of the centre of force.

Table 2 presents the descriptive statistics of occlusal force and occlusion time for the study sample, categorised according to skeletal divergence, along with the Kolmogorov-Smirnov normality test. The occlusion time, obtained from the T-Scan system, was calculated as the time interval between the first occlusal contact during mandibular closure and achievement of maximum intercuspation. Table 3 reports the values of the centre of force position, categorisation of the COF as anterior, or posterior and results from the Kolmogorov-Smirnov normality test.

**Table 2:** Descriptive statistics of occlusal force and occlusion time for the sample categorized by skeletal divergence.

	Normodivergent(*n* = 32)		Hyperdivergent(*n* = 32)		Hypodivergent(*n* = 32)	
	Force	Timing	Force	Timing	Force	Timing
Mean	455.91	7.74	399.54	6.93	513.3	7.9
Median	456.17	8	395.67	6.92	500.33	7.29
Standard deviation	90.2	1.38	122.03	0.89	125.32	1.54
Minimum	348	5.68	227.67	5.66	300.67	5
Maximum	667	10.35	709	8.85	699.33	10.21
Passed normality test	Yes	Yes	Yes	Yes	Yes	Yes

**Table 3:** Descriptive statistics of center of force position categorized as anterior or posterior expressed in percentage.

Center of force	Normodivergent		Hyperdivergent		Hypodivergent	
	Ant. (%)	Post. (%)	Ant. (%)	Post. (%)	Ant. (%)	Post. (%)
Mean	48.56	51.43	23.59	76.41	70.07	29.93
Median	47.9	52.1	23.4	76.6	68.9	31.1
Standard deviation	3.84	3.84	4.35	4.35	5.06	5.06
Minimum	42.3	44.3	14.9	69.1	60.7	20.4
Maximum	55.7	57.7	30.9	85.1	79.6	39.3
Passed normality test	Yes	Yes	Yes	Yes	Yes	Yes

Because the variances were not homogeneous, a Welch ANOVA test was performed, followed by a Games–Howell post hoc test, in order to compare total occlusal force and occlusion time among the groups (Tables 4 and 5). The Holm-Bonferroni correction for multiple testing was also performed. Finally, the association between total occlusal force and centre of force position was analysed using a generalized linear model (GLM) with Gamma distribution and log link function. Total occlusal force was entered as the dependent variable, and centre of force position as a categorical independent variable (Tables 6 and 7). Statistical significance was set at *p* < 0.05. No missing data were recorded for the variables of interest.

**Table 4:** Welch-Anova test for the total force and the occlusion time between the group.

	Statistic	df1	df2	*p*
Total force	15.439	2	129.243	0.000**
Occlusion time	14.640	2	127.083	0.000**

**p < 001.

**Table 5:** Games-Howell’s post hoc test and Holm-Bonferroni correction for multiple tests.

Dependent variable	Group (I)	Group (J)	Mean difference (I-J)	Std.error	Sig.	Holm-Bonferroni correction	Lower bound (CI 95%)	Upper bound (CI 95%)
Total force	Normo	Hyper	54.607**	16.736	0.004	0.012	14.926	94.288
	Hypo	Normo	62.169**	19.779	0.006	0.012	15.186	109.152
	Hypo	Hyper	116.776**	21.143	0.000	0.0000	66.627	166.925
Occlusion time	Normo	Hyper	0.956**	0.192	0.000	0.000	0.500	1.412
	Hypo	Normo	−0.079	0.261	0.95	0.95	−0.700	0.540
	Hypo	Hyper	0.876*	0.245	0.002	0.008	0.293	1.460

**p < 001.

**Table 6:** Results of the generalized linear model (GLM) assessing the effect of center of force position on total occlusal force.

Origin	Type III		
	Wald Chi-square	df	Sig.
(Intercept)	134399.809	1	0.000
Center of force	37.048	2	0.000

Dependent variable: total force. Model: (Intercept), center of force

**Table 7:** Pairwise comparison of the center of force position categorized as anterior, posterior, and centered.

Center of force (I)	Center of force (J)	Mean difference (I-J)	Std. errr	df		Sig.	Lower bound (CI 95%)	Upper bound (CI 95%)
Centered	Posterior	54.607^**^	18.062		1	0.003	19.204	90.01
Anterior	Centered	62.169^**^	20.593		1	0.003	21.806	102.531
Anterior	Posterior	116.776^**^	19.467		1	0.000	78.62	154.933

**p < 001.

## Results

All eligible subjects agreed to participate in the study, and no dropouts were recorded during data collection. Preliminary analyses showed no statistically significant differences between males and females in the main outcome variables. Accordingly, sex was not retained as a relevant factor in subsequent analyses, and the sample was analysed as a single cohort because the distribution of males and females was homogeneous across the analysed groups.

Table 2 highlights a significant finding: the hypodivergent group exhibited the highest total occlusal force and occlusion time (513.3 N and 7.9 s, respectively), while the hyperdivergent group showed the lowest values (399.54 N and 6.93 s) for these parameters. Moreover, Table 3 shows that in the hypodivergent group, the centre of force was predominantly located anteriorly (70.7%), whereas in the hyperdivergent group, the COF was found to be mainly positioned posteriorly (76.41%). Figure 2 illustrates the mean total occlusal force among the three groups.

Differences in total force and occlusion time between groups were found to be statistically significant (both *p* < 0.001); and post hoc pairwise comparisons using the Games–Howell test (Table 5) revealed significant differences in total occlusal force among the groups. Specifically, normodivergent patients demonstrated a total occlusal force that was 54.61 N higher than that measured for the hyperdivergent group (*p* < 0.05). Moreover, hypodivergent patients exhibited significantly greater forces compared to both normodivergent and hyperdivergent groups, with increases of 62.17 N and 116.78 N, respectively (*p* < 0.01). In addition to occlusal force differences, occlusion time durations for normodivergent patients were also significantly longer, by 0.96 s, than for hyperdivergent patients, while hypodivergent patients showed a 0.88 s increase in occlusion time duration relative to the hyperdivergent group (*p* < 0.05).

Application of a generalised linear model revealed a statistically significant association between total occlusal force and the position of the centre of force (*p* < 0.001). Regarding sex distribution, no statistically significant differences were observed (*p* = 0.176). Pairwise comparisons (Table 7) further indicate that individuals with a centred COF exhibited a total occlusal force 54.61 N greater than those with a posteriorly located centre of force (*p* < 0.05). Moreover, study subjects with an anteriorly localised centre of force demonstrated the highest occlusal force values, with an increase of 116.78 N compared to those with a posterior COF (*p* < 0.001). Therefore, both the primary and secondary null hypothesis were rejected.

## Discussion

The present study explored the relationships between occlusal force and the position of the centre of force in adult patients. This interaction is clinically relevant in orthodontics and related disciplines such as prosthodontics and gnathology, because it may help inform the planning and execution of therapeutic strategies. In modern populations, the force required to chew most foods is substantially lower than the maximum voluntary bite force. However, maximum occlusal force reflects the biomechanical and neuromuscular capacity of the craniofacial system rather than routine masticatory demands. Although not fully utilized during daily chewing, it has been suggested that it may be related to craniofacial development and morphology.

Bite force is known to vary with the degree of gape. However, in the present study, force recordings were performed using a mouthpiece of standardized thickness, ensuring a consistent degree of gape across all participants. Variations in the fluid within the device were dependent solely on the magnitude of the applied biting force. Additionally, all subjects were adults with stable neuromuscular and skeletal characteristics (CS5), minimizing potential confounding effects related to growth.

Detailed data analysis revealed statistically significant associations between craniofacial morphology and functional occlusal parameters, such as occlusion time and total occlusal force. Total occlusal force was significantly greater in the hypodivergent group (513.3 N), followed by the normodivergent group (455.91 N), while hyperdivergent subjects exhibited the lowest occlusal force values in the study sample (399.54 N). These findings are consistent with the biomechanical characteristics previously described for each group. For example, hypodivergent individuals are characterised by a compact craniofacial morphology and an anteriorly rotated mandible with a relatively horizontal mandibular plane, and have been reported to exhibit enhanced masticatory efficiency^25^. This pattern has been associated with greater functional capacity and hypertrophy of the masseter and temporalis muscles^26,27^. In contrast, hyperdivergent individuals typically present with a long-face skeletal pattern and reduced masticatory muscle development, features that have been associated with lower masticatory efficiency and reduced occlusal force values.

Occlusion time at maximum intercuspation was also associated with vertical skeletal divergence, and statistically significant differences were identified among the groups. Specifically, hypodivergent individuals had the longest occlusion times and hyperdivergent subjects had the shortest occlusion times, while intermediate values were observed in normodivergent patients. This pattern parallels the total occlusal force distribution observed in each group and supports an association between craniofacial morphology and functional occlusal dynamics. Analysis of total occlusal force and occlusion time also revealed that higher occlusal force was associated with longer occlusion time to reach maximum intercuspation. This finding may reflect differences in mandibular closure pattern and neuromuscular coordination under greater occlusal load. The present findings are in agreement with previous evidence indicating greater occlusal stability among hypodivergent individuals. As reported by Gomes et al.^28^, hypodivergent patients typically exhibit more stable and evenly distributed occlusal contacts. The association between increased occlusal force and prolonged occlusion time in hypodivergent subjects may reflect a more controlled mandibular closure pattern. In contrast, individuals with increased vertical skeletal dimensions displayed faster occlusal dynamics together with lower occlusal forces, a pattern that may be indicative of reduced occlusal stability and less efficient neuromuscular function^29,30^. An important finding of the present study was the significant association between the location of the centre of force and total occlusal force. Participants exhibiting an anteriorly positioned centre of force showed significantly greater occlusal forces than those with a central or posteriorly localised centre of force. This observation suggests that an anteriorly directed occlusal load may be associated with more favourable masticatory function, possibly because of enhanced intercuspation and more balanced load distribution across the dental arches. In contrast, subjects with a posteriorly localised centre of force showed reduced occlusal force values, which may reflect lower functional efficiency and less stable occlusal contact distribution^31^.

Hypodivergent subjects exhibited a more anteriorly positioned centre of force together with higher occlusal force values. Their skeletal-muscular configuration may provide mechanical advantages to the masticatory muscles, allowing more efficient function and a greater capacity to generate occlusal force. This pattern may be related to a more compact facial structure, often characterised by a shorter mandibular ramus and reduced vertical dimension, which could favour a more efficient distribution of occlusal loads^32^. In contrast, hyperdivergent individuals exhibited a more posterior centre of force associated with lower occlusal force values. This morphological pattern may be related to a lower capacity to generate high-intensity occlusal forces. In hyperdivergent subjects, mandibular retrusion combined with increased vertical dimension and posterior mandibular rotation may be associated with reduced occlusal stability and a narrower, less balanced distribution of occlusal loads^33^.

Biomechanically, the mandible can be considered a third-class lever, where the force is applied between the fulcrum and the resistance. The fulcrum corresponds to the temporomandibular joint, while the force is generated at the insertion point of the masticatory muscles and the resistance is represented by the occlusal load^34,35^. Although the mandible maintains the characteristics of a third-class lever, some anatomical features typical of hypodivergent individuals may be associated with more favourable mechanical conditions. In this context, a more anteriorly positioned occlusal load may increase the functional efficiency of the elevator muscles. This morphotype is typically characterised by reduced vertical facial dimension and a shorter, more robust mandibular ramus, features that may be associated with greater neuromuscular efficiency and higher resistance to fatigue. Conversely, hyperdivergent subjects are often characterised by longer and thinner bony bases, with increased vertical dimensions and altered spatial relationships between the temporomandibular joint, muscle insertion points, and occlusal load. This configuration may be mechanically less advantageous and may be associated with greater muscular effort during function. These biomechanical differences may partly explain the lower occlusal force values observed in hyperdivergent subjects. However, this configuration allows for wider and faster movements. Consequently, to lift a load (resistance) and apply the necessary force for movement, the masticatory muscles must generate greater force, resulting in increased energy expenditure. This dynamic has direct neurophysiological implications, because hyperdivergent individuals must apply greater muscular effort. This leads to increased recruitment of motor units within type II muscle fibres (which are stronger but less fatigue-resistant) to compensate for the biomechanical disadvantage. The consequent overload of type II muscle fibres contributes to the overall reduced occlusal force and earlier onset of neuromuscular fatigue.

Application of the generalized linear model showed that occlusal force was significantly associated with the position of the centre of force. Specifically, the highest force values were observed in individuals with an anteriorly positioned centre of force, whereas the lowest occlusal force values were found in subjects with a posteriorly positioned centre of force.

The variation in occlusal force according to the position of the centre of force and craniofacial morphology may be interpreted in light of the neurophysiological and biomechanical mechanisms of the masticatory muscles, particularly those involving motor unit recruitment. The force generated by a muscle depends on the number of motor units activated and the frequency of their discharge. According to Henneman’s size principle, motor units are recruited in an orderly fashion from the smallest to the largest, based on increasing threshold^36,37^. Smaller motor units are associated with lower-threshold motor neurons and typically innervate type I slow-twitch muscle fibres, which are highly fatigue-resistant and are recruited during low-intensity sustained activities. As force demand increases, larger motor units with high-threshold motor neurons are progressively recruited. These innervate type II fast-twitch muscle fibres, which are more susceptible to fatigue^38^. A similar graded recruitment pattern has also been proposed for the masticatory muscles. It has been suggested that hypodivergent individuals may recruit a greater proportion of type II muscle fibres, whereas hyperdivergent individuals may rely more heavily on type I fibres. However, these mechanisms remain hypothetical in the context of the present study and should be interpreted cautiously.

Maximum occlusal force is a multifactorial parameter influenced not only by craniofacial morphology but also by several biological and behavioural variables. Body mass index has been reported to correlate positively with occlusal force, likely reflecting greater overall muscle mass and an increased cross-sectional area of the masticatory muscles^13,39^. Similarly, masticatory muscle thickness, particularly of the masseter and temporalis muscles, plays a key role in force generation, as greater muscle thickness is associated with enhanced neuromuscular recruitment and higher force-producing capacity^26,40^. Parafunctional habits such as bruxism and clenching may further modulate occlusal force by inducing adaptive changes in muscle activity and morphology^41^; depending on individual neuromuscular response, these habits may result either in increased maximal force due to muscle hypertrophy or in reduced efficiency secondary to fatigue and altered motor control^10^. In addition, sex-related differences in occlusal force have been widely reported and are generally attributed to differences in muscle mass and hormonal influences. However, these differences tend to be less pronounced in homogeneous adult samples with completed craniofacial growth. Nevertheless, in the present study no statistically significant sex-related differences were detected. This finding may be attributed to comparable lifestyle patterns, genetic backgrounds, and similar dietary habits within the analyzed sample, which may have contributed to minimizing sex-related variability.

The data obtained in the present study indicate that a hypodivergent craniofacial morphotype is associated with a configuration that may optimise masticatory biomechanics, with greater neuromuscular efficiency and a higher capacity to generate occlusal forces.

From a clinical perspective, the present findings highlight the importance of considering craniofacial morphology when evaluating occlusal function. Differences in occlusal force magnitude, occlusion time, and centre of force position among vertical skeletal patterns suggest that hypodivergent and hyperdivergent patients exhibit distinct functional and biomechanical characteristics. Incorporating digital occlusal analysis into orthodontic diagnosis may provide valuable information beyond static occlusal relationships, allowing clinicians to better identify functional imbalances and adapt treatment strategies accordingly. In particular, assessment of occlusal force distribution and centre of force position may support more individualized treatment planning, potentially improving functional stability and reducing the risk of occlusal overload or dysfunction following orthodontic therapies.

### Limitations of the study

The absence of electromyographic analysis of the masticatory muscles represents a limitation, and other variables known to influence occlusal force, such as body mass index or muscle thickness, were not specifically evaluated. Although the study had a prospective design, causal inferences cannot be fully established. Moreover, the inclusion of only young adult subjects with Angle Class I malocclusion limits the generalizability of the findings. Potential device-related variability between the T-Scan and Innobyte systems should also be considered, as differences in measurement principles may have affected the recorded occlusal force values. In addition, occlusal force was recorded at a single time point, which may not fully reflect physiological variations over time. Future studies including larger and more diverse populations, repeated occlusal force assessments, and integrated electromyographic analyses are needed to confirm and extend the present findings.

## Conclusions

Hypodivergent individuals have a biomechanical advantage, characterised by higher occlusal forces and prolonged contact times, in contrast, hyperdivergent subjects have diminished occlusal force and altered contact dynamics. Normodivergent individuals represent an intermediate phenotype, maintaining balanced occlusal function and force distribution. These findings suggest that considering craniofacial morphology and centre of force analyses may contribute to more complete diagnosis and treatment planning in occlusal and orthodontic therapies, potentially supporting improved functional outcomes and minimising dysfunction.

## Data Availability

The data supporting the findings are available from the corresponding author upon reasonable requests.
